# A rat model for hepatitis E virus

**DOI:** 10.1242/dmm.024406

**Published:** 2016-10-01

**Authors:** Yannick Debing, Niraj Mishra, Erik Verbeken, Kaat Ramaekers, Kai Dallmeier, Johan Neyts

**Affiliations:** 1 Rega Institute for Medical Research, Department of Microbiology and Immunology, KU Leuven, Leuven 3000, Belgium; 2 Department of Imaging & Pathology, Translational Cell & Tissue Research, KU Leuven, Leuven 3000, Belgium

**Keywords:** Hepatitis E virus, Rat, Animal model, Antiviral, Ribavirin, LA-B350

## Abstract

Hepatitis E virus (HEV) is one of the prime causes of acute viral hepatitis, and chronic hepatitis E is increasingly recognized as an important problem in the transplant setting. Nevertheless, the fundamental understanding of the biology of HEV replication is limited and there are few therapeutic options. The development of such therapies is partially hindered by the lack of a robust and convenient animal model. We propose the infection of athymic nude rats with the rat HEV strain LA-B350 as such a model. A cDNA clone, pLA-B350, was constructed and the infectivity of its capped RNA transcripts was confirmed *in vitro* and *in vivo*. Furthermore, a subgenomic replicon, pLA-B350/luc, was constructed and validated for *in vitro* antiviral studies. Interestingly, rat HEV proved to be less sensitive to the antiviral activity of α-interferon, ribavirin and mycophenolic acid than genotype 3 HEV (a strain that infects humans). As a proof-of-concept, part of the C-terminal polymerase sequence of pLA-B350/luc was swapped with its genotype 3 HEV counterpart: the resulting chimeric replicon replicated with comparable efficiency as the wild-type construct, confirming that LA-B350 strain is amenable to humanization (replacement of certain sequences or motifs by their counterparts from human HEV strains). Finally, ribavirin effectively inhibited LA-B350 replication in athymic nude rats, confirming the suitability of the rat model for antiviral studies.

## Abstract (precis)

**Summary:** Rat hepatitis E virus strain LA-B350 is used as a model for antiviral studies for hepatitis E virus using a cDNA clone, replicon and *in vivo* studies.

## INTRODUCTION

Hepatitis E virus (HEV) is a positive-sense single-stranded RNA virus classified in the *Hepeviridae* family. The virus is transmitted feco-orally and, although most infections remain asymptomatic, HEV is one of the most common causes of acute viral hepatitis and is known to cause large water-borne epidemics in developing countries (Debing and Neyts, 2014; Wedemeyer et al., 2013). Such outbreaks are mainly due to infections with genotype 1 and are typically associated with high mortality rates in pregnant women (Khuroo et al., 1981; Tsega et al., 1993). Genotypes 3 and 4 are the main causes of hepatitis E in industrialized countries, where infections occur mostly through consumption of undercooked pig meat (Dalton et al., 2014). Genotype 3 infections may evolve to chronicity in immunocompromised individuals (e.g. transplant recipients, HIV-infected individuals etc.) and often require either dose reductions of immunosuppressant drugs and/or treatment with ribavirin or pegylated α-interferon (Kamar et al., 2011). Ribavirin is the drug of choice in most patients (Kamar et al., 2014), but has several side effects and treatment failure occurs occasionally (Debing et al., 2014d; Pischke et al., 2013). A robust and convenient animal infection model for hepatitis E will allow us to further explore the modalities for antiviral therapy and to develop alternative treatment options for (chronic) hepatitis E. Currently available infection models such as those in chimpanzees and pigs are not convenient, whereas attempts to infect rodents with human HEV strains generated mixed outcomes and did not result in a robust animal model (Huang et al., 2009; Li et al., 2008, 2013a,b; Purcell et al., 2011).

Rat HEV is another member of the *Hepeviridae* family and has been isolated from wild rats and probably has a worldwide prevalence (Johne et al., 2010b; Mulyanto et al., 2014; Purcell et al., 2011). Rats seem to acquire the virus at an early age and infection generally proceeds without overt symptoms (Purcell et al., 2011). Rat HEV is classified in the *Orthohepevirus* C species according to recently proposed HEV taxonomy recommendations (Smith et al., 2014), whereas human genotypes 1-4 are grouped under the *Orthohepevirus* A species. Furthermore, rat HEV strains are classified into three different genetic groups (Mulyanto et al., 2014). Sequence identity between human and rat HEV is about 60%, with several highly conserved motifs in key viral genes.

Because rat HEV can infect several types of laboratory rats (Li et al., 2013a; Purcell et al., 2011), we aimed to employ this virus in a *bona fide* model for infections with *Hepeviridae*. The rat HEV strain LA-B350 was originally isolated from rats in Los Angeles, CA, and was subsequently passaged in Sprague-Dawley rats and in an athymic nude hooded rat (B350) (Purcell et al., 2011). We recently determined the full-length genomic sequence of this strain (Debing et al., 2014a). Here, we describe the establishment of the pLA-B350 cDNA clone and the corresponding subgenomic replicon pLA-B350/luc, and confirm the suitability of these constructs for antiviral studies.

## RESULTS

### Athymic nude rats, but not mice, are susceptible to rat HEV LA-B350 infection

**Fig. 1.**
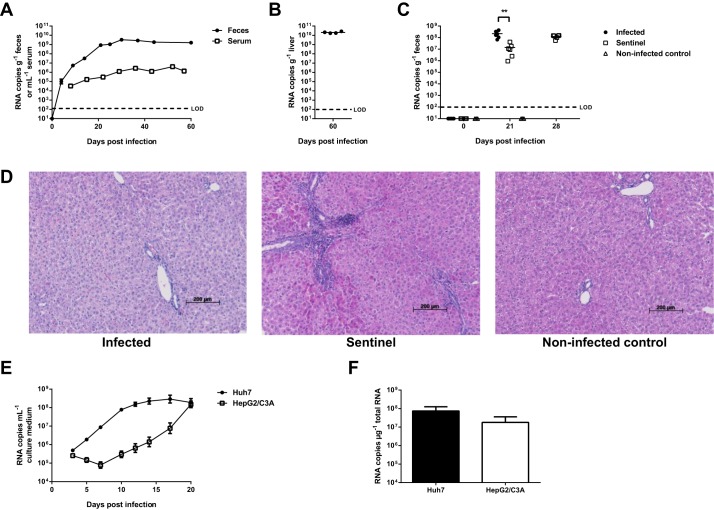
**Rat HEV strain LA-B350 is infectious *in vivo* and *in vitro*.** Four athymic nude rats were inoculated intravenously with LA-B350 and virus quantities in feces and serum (A) and liver (B) were determined by RT-qPCR, documenting robust infection. In a subsequent transmission experiment, LA-B350-infected rats were co-housed with non-infected sentinels. Sentinels displayed robust shedding of virus in stool after 21 and 28 days, indicating efficient transmission of rat HEV (C). Hematoxylin-eosin staining of liver sections showed only very limited infiltration of portal tracks with lymphocytes and polymorphonuclear leucocytes in infected animals (D). LA-B350 also proved infectious *in vitro* in Huh7 and HepG2/C3A cells: viral RNA released in the culture medium increased over time (E) and high amounts of RNA were detected intracellularly after 20 days (F). Values represent mean±s.e.m. for four animals (A) or from three independent experiments (E,F). The limit of detection for panels E and F is approximately 10^3^ RNA copies ml^−1^ culture medium or µg^−1^ total RNA, respectively. LOD, limit of detection. **P<0.01 (two-tailed *t*-test).

To develop a convenient and robust animal model for HEV using rat HEV strain LA-B350, four athymic nude rats were injected intravenously with diluted liver suspension from rat B350 (Purcell et al., 2011); in all four rats, solid viral replication was observed (Fig. 1A,B). Rat HEV RNA was detected in the feces as early as 4 days post-infection and increased to over 10^9^ copies g^−1^ feces by day 30; afterwards, fecal titers remained constant until day 60 post-infection. Viral RNA was detected in rat serum as well, although at considerably lower titers compared to the feces, reaching a maximum of 4×10^6^ copies ml^−1^ serum (Fig. 1A). After 60 days, viral RNA levels in the liver reached over 10^10^ copies g^−1^ liver (Fig. 1B). These titers are higher than those reported by others (Li et al., 2013a; Purcell et al., 2011), which may be the result of the extended time frame and/or adaptation to the host in the current study. No marked alterations were observed in levels of alanine aminotransferase (ALT), aspartate aminotransferase (AST) or γ-glutamyltransferase (γGT) in serum (Fig. S1). Concordantly, histopathological evaluation of hematoxylin- and eosin-stained liver sections revealed only very limited evidence for portal tract infiltration by lymphocytes and polymorphonuclear leucocytes in infected animals, with no significant differences compared to uninfected control rats (Fig. 1D). This lack of hepatic injury in rat HEV-infected animals is in line with earlier reports (Li et al., 2013a; Purcell et al., 2011).

To study whether the virus is efficiently transmitted to sentinel rats, sentinel animals were co-housed together with rats that had been infected with strain LA-B350. Following 21 days of co-housing, all sentinels were found to be infected. High titers of viral RNA were detected in the feces of these animals, although these titers were lower than those of the originally infected rats (Fig. 1C). Viral titers further increased until day 28 after the beginning of the co-housing. Overall, these data confirm efficient transmission of rat HEV between co-housed animals.

We also attempted to infect several mouse strains with the LA-B350 rat HEV strain. Although no viral RNA could be detected in the feces or liver of intravenously injected severe combined immunodeficient (SCID) and athymic nude mice, low levels of rat HEV RNA were detected in three out of three livers of inoculated AG129 mice (about 10^6^ copies g^−1^ liver), but not in the feces (data not shown). Attempts to passage the virus by injecting other AG129 mice with liver homogenates from these three infected mice were unsuccessful, however.

### Human hepatoma cell lines Huh7 and HepG2/C3A support solid LA-B350 replication

Next, several hepatoma cell lines were inoculated with LA-B350 rat HEV obtained from day-60 rat livers. In line with earlier findings (Jirintai et al., 2014), solid replication was observed in Huh7 and HepG2/C3A cell lines (Fig. 1E,F). Replication in HepG2/C3A cells was somewhat delayed when compared to Huh7 cells. In contrast to the findings of Jirintai et al. (2014), we could not detect any replication in PLC/PRF/5 hepatoma cells. The rat hepatoma cell line H4IIE did not support rat HEV replication either (data not shown).

### Construction and *in vitro* testing of cDNA clone pLA-B350

**Fig. 2.**
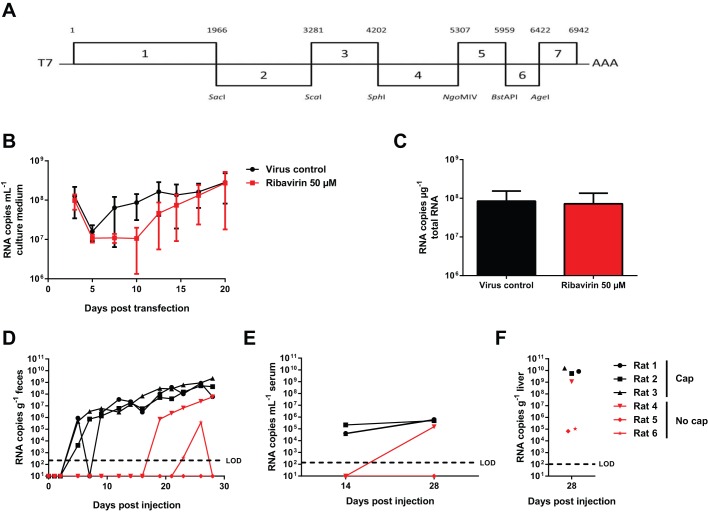
**RNA transcripts from cDNA clone pLA-B350 induce robust infection *in vitro* and *in vivo*.** A cDNA clone for LA-B350 was constructed based on the consensus sequence from seven overlapping fragments (A). Capped pLA-B350 transcripts were transfected into Huh7 cells and induced robust infection (B,C). Intrahepatic injection of capped pLA-B350 RNA in athymic nude rats resulted in robust shedding of rat HEV RNA in the feces (D) and serum (E), whereas high amounts of virus were detected in the liver after 28 days (F). Injection of non-capped RNA only induced successful infection in 1/3 rats. Values represent mean±s.e.m. from three independent experiments (B,C) or individual values for each rat (D-F). LOD, limit of detection.

To develop rat HEV LA-B350 as a convenient tool for fundamental and antiviral studies, a full-length cDNA clone was constructed of the LA-B350 consensus sequence (pLA-B350; Fig. 2A). Transfection of a capped RNA transcript in Huh7 cells confirmed the *in vitro* replication competence of the pLA-B350 construct, with increasing viral titers over 20 days (Fig. 2B). The rather high number of RNA copies at day 3 post-transfection probably represents RNA remnants of the initial inoculum. Interestingly, the addition of ribavirin to a concentration of 50 µM resulted only in a delay in the increase of viral RNA in the culture medium, yielding equally high amounts of viral RNA at day 20 post-transfection, both in culture medium and intracellularly (Fig. 2B,C). This is in contrast with genotype 3 human HEV replication, which is strongly inhibited by ribavirin at 50 µM (Debing et al., 2014b).

### Intrahepatic inoculation of capped pLA-B350 RNA transcripts results in robust infection and shedding of infectious virus

**Fig. 3.**
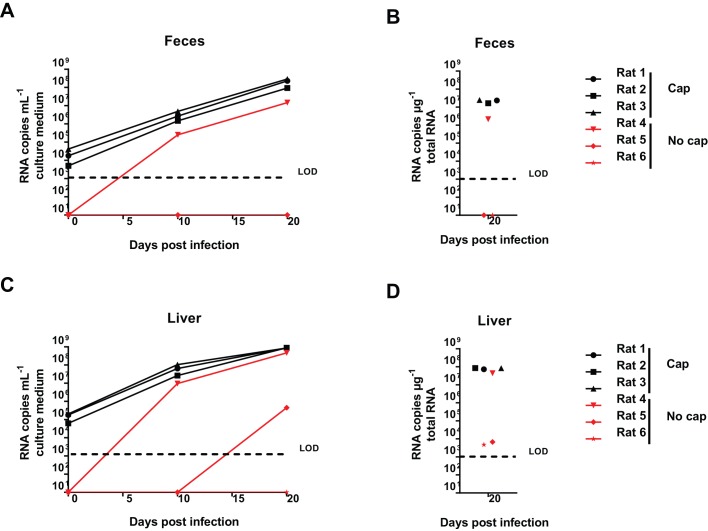
**Virus progeny from pLA-B350 RNA-injected rats is infectious in cell culture.** Huh7 cell cultures were inoculated with feces (A,B) or liver (C,D) suspensions from pLA-B350 RNA-injected rats (28 days post-infection). Robust viral replication was observed for cultures corresponding to rats 1-3 (capped) and rat 4 (non-capped). Values represent individual values for each culture/rat. LOD, limit of detection.

Next, we assessed the *in vivo* replication potential of pLA-B350 by injecting capped pLA-B350 RNA transcripts intrahepatically in three athymic nude rats. Viral RNA could be detected in the feces 4 days after inoculation and titers increased rapidly over the following 24 days (Fig. 2D), in a similar fashion as observed for rats injected with infectious LA-B350 (Fig. 1A). As a negative control, three other rats were injected with non-capped transcripts: robust viral shedding was observed in only one of these rats, although with a clear delay compared to rats injected with the capped RNA. Quantification of rat HEV RNA in serum and liver confirmed these findings (Fig. 2E,F).This is in line with an earlier report describing the necessity of capping for infectivity of genotype 1 HEV RNA in rhesus monkeys (Emerson et al., 2001). To assess whether the viral RNA detected in feces and liver indeed represents infectious virus, Huh7 cell cultures were inoculated with rat HEV from fecal and liver suspensions from pLA-B350 RNA-inoculated rats at day 28 post-injection: release of viral RNA in the culture medium was assessed at day 10 and 20, and intracellular levels were quantified at day 20 (Fig. 3). Cultures inoculated with fecal extracts from rats 1-4, but not rats 5 and 6, resulted in efficient viral replication (Fig. 3A,B), whereas viral replication was detected in cultures that had been inoculated with liver extracts of rats 1-5 and intracellular rat HEV RNA could be detected for all rats (Fig. 3C,D), suggesting that a limited amount of infectious virus might have been produced in the livers of rats 5 and 6 as well. Taken together, these data confirm the infectivity of pLA-B350.

### Subgenomic replicon pLA-B350/luc as a tool for antiviral drug testing

**Fig. 4.**
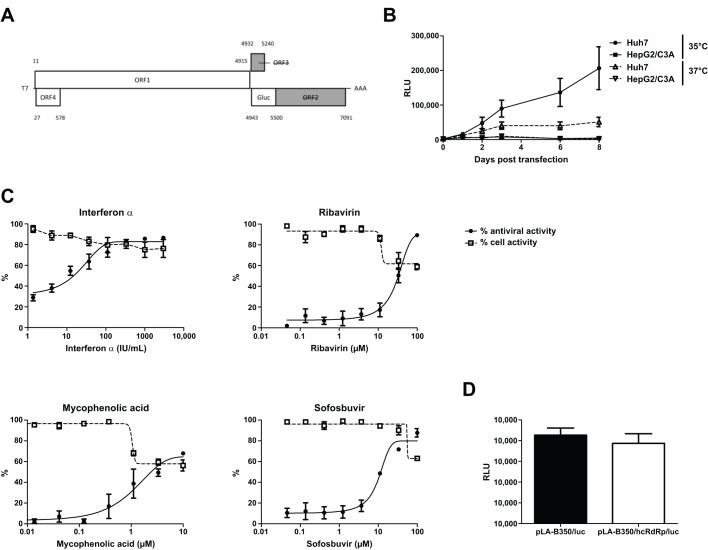
**Subgenomic replicon pLA-B350/luc is suitable for antiviral testing and is amenable to humanization.** The pLA-B350/luc subgenomic replicon was constructed by inserting a *Gaussia* luciferase reporter gene downstream of the ORF2 start codon (A). pLA-B350/luc displayed strong viral replication in Huh7 cells at 35°C, but less at 37°C, whereas HepG2/C3A yielded very low luminescence signals, probably due to transfection toxicity (B). Dose–response curves represent antiviral effects and cell viability for α-interferon, ribavirin, mycophenolic acid and sofosbuvir (C). Part of the C-terminal RdRp sequence of pLA-B350/luc was replaced with its genotype 3 HEV counterpart, without affecting viral replication (D). Values represent mean±s.e.m. from three independent experiments. RLU, relative light units.

When using rat HEV as an *in vivo* model for HEV, it would be highly convenient to have a tool that allows preliminary *in vitro* testing of potential antiviral drugs on rat HEV before animal testing. To this end, the subgenomic replicon pLA-B350/luc was constructed, in which a *Gaussia* luciferase reporter gene was inserted at the ORF2 start codon (Fig. 4A), similarly to the HEV genotype 3 Kernow-C1 p6/luc replicon (Shukla et al., 2012). Capped pLA-B350/luc RNA was transfected into Huh7 and HepG2/C3A hepatoma cells: solid replication was observed in Huh7 cells cultured at 35°C, whereas luminescence was considerably lower at 37°C (Fig. 4B), in line with findings for human HEV strains (Shukla et al., 2012; Takahashi et al., 2007). Transfection of HepG2/C3A cells yielded very low luminescence values, probably due to transfection toxicity – a phenomenon that we observed earlier for the genotype 1 Sar55/S17/luc replicon in these cells (data not shown). Next, pLA-B350/luc was employed in a 3-day antiviral assay in Huh7 cells (Fig. 4C). Interferon-α potently inhibited the replicon, with an EC_50_-value of 10±5 international units (IU) ml^−1^, whereas the activity of ribavirin was less pronounced (EC_50_ of 33±9 µM). Interestingly, the activities of both compounds are approximately tenfold weaker as those observed for the genotype 3 Kernow-C1 p6/luc replicon in the same cell type (Debing et al., 2014b). In line with results obtained for ribavirin, the activity of mycophenolic acid, another GTP-depleting drug, was limited (EC_50_ of 3±2 µM, with some toxicity). We also tested sofosbuvir, which is a potent inhibitor of the hepatitis C virus RNA-dependent RNA polymerase (RdRp) (Sofia et al., 2010) and also exhibits some *in vitro* antiviral activity against HEV (Dao Thi et al., 2016). Sofosbuvir inhibited pLA-B350/luc replication, with an EC_50_ of 12±1 µM, which is very much comparable to the inhibition observed in the HEV p6/luc system (Dao Thi et al., 2016).

### Partial humanization of the rat HEV RdRp

To study the role of specific motifs in HEV non-structural proteins and to increase the relevance of the rat HEV system, it would be highly useful for the construct/replicon to be amenable to humanization, i.e. replacement of certain sequences or motifs by their counterparts from human HEV strains. As a proof-of-concept, we replaced the DFVAKLRNV motif at the RdRp C-terminus of pLA-B350/luc with the corresponding DFLRGLTNV sequence from HEV genotype 3, resulting in four amino-acid changes. This specific region was selected given the role of the G1634R mutation in ribavirin treatment failure (Debing et al., 2014d). The resulting chimeric pLA-B350/hcRdRp/luc construct replicated to a similar extent as the wild-type pLA-B350/luc replicon (Fig. 4D, *P*=0.52, *t*-test), illustrating the possibilities for further humanization of the rat HEV system.

### Ribavirin inhibits LA-B350 replication *in vivo*

**Fig. 5.**
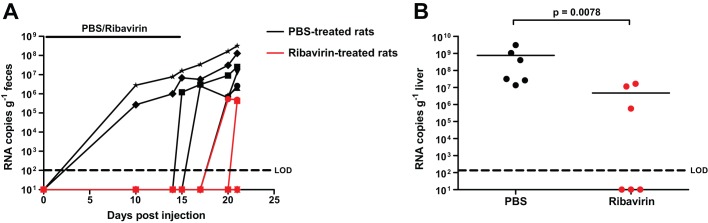
**Ribavirin treatment of LA-B350-infected rats delays virus shedding and significantly reduces viral loads in the liver.** Twelve rats were infected intravenously with rat HEV LA-B350. Afterwards, six of these rats were injected daily with ribavirin at 30 mg/kg body weight (red), whereas the other six were injected with PBS (black), for 14 days. Rat HEV was quantified in feces (A) and in liver 21 days after infection (B). Values represent individual values for each rat; significance was calculated through the Mann–Whitney *U*-test (B). LOD, limit of detection.

Finally, we assessed the suitability of the rat model for *in vivo* antiviral studies. LA-B350-infected rats were treated intraperitoneally with either phosphate-buffered saline (PBS) or ribavirin at 30 mg kg body weight^−1^ day^−1^ for 14 days after infection, followed by 7 days without treatment before sacrifice. We detected robust virus shedding in the feces in 6/6 PBS-treated rats, but only in 2/6 ribavirin-treated rats (Fig. 5A). Similarly, viral RNA was detected in all PBS-treated rat livers, but only in 3/6 livers of the ribavirin group. Overall, significantly more rat HEV RNA was found in the livers from the PBS group compared to the ribavirin group: 7.6×10^8^ vs 4.7×10^7^ RNA copies g^−1^ liver (Fig. 5B; *P*=0.0078, Mann–Whitney *U*-test), confirming the efficacy of ribavirin treatment in the rat HEV model, despite a limited *in vitro* antiviral effect.

## DISCUSSION

HEV is estimated to cause ∼56,600 deaths each year and chronic hepatitis E is increasingly recognized as an important problem in the transplant setting (Sayed et al., 2015; WHO, 2015). Nevertheless, our understanding of HEV biology is still very limited and therapeutic options are few (Debing and Neyts, 2014). This is partially due to the lack of a convenient and robust animal model: most *in vivo* studies to date relied on the use of monkeys or pigs (Yugo et al., 2014). Rat HEV represents an interesting opportunity to fill this gap: the virus is closely related to human/swine HEV and readily infects laboratory rats (Li et al., 2013a; Purcell et al., 2011). Therefore, we developed the necessary tools to use rat HEV strain LA-B350 as a *bona fide* animal model for HEV. LA-B350 readily infects athymic nude rats, in which it causes a chronic infection, which resembles the situation in immunocompromised individuals with chronic hepatitis E. A full-length cDNA clone was constructed from the LA-B350 consensus sequence and the infectivity of its RNA transcripts was shown *in vitro* and *in vivo*. Furthermore, we constructed the pLA-B350/luc subgenomic replicon, which is suitable for *in vitro* antiviral testing. This allows easy testing of anti-rat HEV activity of a molecule before initiating animal studies. Finally, a proof-of-concept was provided for the humanization of parts of the rat HEV sequence.

The construction of a cDNA clone of rat HEV strain R63/DEU/2009 was recently reported (Li et al., 2015). Contrary to the cDNA clone of this previous study, which contains 23 mutations compared to the original sequence, pLA-B350 is based on the consensus sequence of the LA-B350 strain and any PCR-induced mutations were corrected. LA-B350 also has the advantage of replicating in the commonly used Huh7 and HepG2/C3A hepatoma cell lines, but we could not detect solid replication in PLC/PRF/5 cells, contrary to the results for R63/DEU/2009 (Li et al., 2015).

An early report on rat HEV indicated a lack of replication in rat liver cell lines such as N1-S1, clone 9, and MH1C1 (Johne et al., 2010a). In addition, we were unable to culture LA-B350 in H4IIE rat hepatoma cells. Conversely, and surprisingly, human hepatoma cell lines such as Huh7, HepG2/C3A and PLC/PRF/5 support solid rat HEV replication (Jirintai et al., 2014). Although the virus was not transmissible to rhesus monkeys (Purcell et al., 2011), rat HEV-specific antibodies have been found in forestry workers (Dremsek et al., 2012). This raises questions about the zoonotic potential of rat HEV. The tools developed in this study might contribute to elucidating rat HEV's role as a zoonotic pathogen.

Another interesting observation is the reduced sensitivity of pLA-B350/luc to the antiviral effects of α-interferon, ribavirin and mycophenolic acid, despite the fact that the same Huh7 cell line was employed as in the earlier genotype 3 HEV studies (Debing et al., 2014b). We showed previously that the antiviral effect of both ribavirin and mycophenolic acid depends on the depletion of intracellular GTP pools (Debing et al., 2014b), suggesting that the rat HEV RdRp might inherently use GTP in a more efficient manner. Despite this reduced sensitivity, ribavirin was still able to significantly lower viral loads *in vivo*, corroborating the use of LA-B350 as an HEV surrogate, even if there are differences in sensitivity between rat and human HEV. Although we demonstrated that sofosbuvir exerts *in vitro* activity against the rat HEV, the potential protective effect of this compound in the rat infection model cannot be studied. Indeed, rats have high levels of esterase activity in the gastrointestinal tract and serum, which results in rapid degradation of the prodrug moiety of sofosbuvir and, as a consequence, in very low amounts of the active 5′-triphosphate in the liver (Dao Thi et al., 2016). However, the rat model will still be highly useful to study the *in vivo* antiviral activity of other antiviral compounds, both established drugs and novel molecules.

Finally, the successful replacement of part of the rat HEV RdRp with a genotype 3 HEV sequence opens up possibilities for further humanization of the RdRp and possibly other viral proteins, making the model more relevant to the human situation. Such chimeras could also be used to study the molecular replication mechanisms of the *Hepeviridae* or to determine viral factors governing host specificity.

## MATERIALS AND METHODS

### Cells and viruses

Huh7 cells (a kind gift from Ralf Bartenschlager, University of Heidelberg, Germany) and HepG2/C3A cells (a kind gift from Luc Verschaeve, Scientific Institute of Public Health, Brussels, Belgium) were cultured in Dulbecco's modified Eagle's medium (DMEM; Gibco, Carlsbad, CA) supplemented with 10% fetal bovine serum (FBS; Gibco) in a humidified 5% CO_2_ incubator at 37°C. Cell lines were regularly tested for Mycoplasma contamination.

Rat HEV strain LA-B350 (GenBank accession no. KM516906) stock was a 10% liver homogenate from rat B350 (kindly provided by Suzanne U. Emerson, National Institute of Allergy and Infectious Diseases, National Institutes of Health, Bethesda, MD) (Debing et al., 2014a; Purcell et al., 2011).

### Construction of the pLA-B350 cDNA clone

Total RNA was extracted from 100 µl of a 10% liver homogenate from rat B350 with the Qiagen RNeasy kit (Hilden, Germany) and rat HEV cDNA was generated using the One-Step reverse transcription PCR kit (Qiagen) and KAPA Hi-Fi HotStart ReadyMix PCR kit (Kapa Biosystems, Wilmington, MA). Primers were designed based on relatively conserved regions in other rat HEV sequences and strain-specific sequences as they became available. Fragments were gel-purified, cloned into the pJet1.2 vector using the Clone-JET PCR cloning kit (Thermo Fisher Scientific, Waltham, MA), and sequenced as described (Debing et al., 2014a). Fragments and restriction enzymes used to assemble the full-length cDNA are depicted in Fig. 2A. First, fragments 6 and 7 were fused, followed by the addition of fragments 5, 4 and 3. Fragments 1 and 2 were fused together and finally fragments 1-2 and 3-7 were ligated. Residues not corresponding to the consensus sequence were mutated to match the consensus. An *Xba*I restriction site and T7 promoter sequence were inserted at the 5′ end and a 80-bp polyA-tail and a *Mlu*I restriction site were added at the 3′ end. Next, the full-length T7-LA-B350-polyA was cloned into pBlueScript SK (+) using *Xba*I and *Mlu*I to yield pLA-B350. *Escherichia coli* Top10 cells (Life Technologies, Carlsbad, CA) were transformed with ligated plasmid and cultured in 100 ml Terrific Broth (Carl Roth, Karlsruhe, Germany) with ampicillin and midiprepped (NucleoBond Xtra midi kit, Macherey-Nagel, Düren, Germany). The entire construct was sequenced and mutations not corresponding to the consensus sequence were corrected. Primer sequences are available upon request.

### Construction of pLA-B350/luc and pLA-B350/hcRdRp/luc replicons

To construct pLA-B350/luc, the *Gaussia* luciferase gene was amplified from the Kernow-C1 p6/luc construct (Shukla et al., 2012), fused to flanking regions of pLA-B350 by fusion PCR, and inserted into pLA-B350 using *Afl*II and *Eco*RI. The *Gaussia* luciferase gene was inserted at the ORF2 starting codon (replacing the sequence between positions 4943 and 5285 of pLA-B350; Fig. 4A).

To replace the DFVAKLRNV motif at the C-terminus of the RdRp (aa 1575-1583 according to AIP98381 numbering) with the corresponding human HEV genotype 3 motif DFLRGLTNV, PCRs were performed on pLA-B350/luc using primer pairs 5′-ATGGTGCTTTTATGGCGATTG-3′+5′-CGTCAACCCTCGAAGAAAGTCGCGCACGGAC-3′ and 5′-CTTCGAGGGTTGACGAATGTCACTGCCTTG-3′+5′-GGCAGCCACTTCTTGAGCAG-3′. Fragments were fused using outer primers and inserted into pLA-B350/luc with *Afl*II and *Nco*I, yielding pLA-B350/hcRdRp/luc.

### *In vitro* transcription and capping

Viral RNA was *in vitro* transcribed from *Mlu*I-linearized plasmid DNA with the RiboMAX Large Scale RNA Production System-T7 (Promega, Madison, WI) and capped with the ScriptCap m7G capping system (Cellscript, Madison, WI). Nucleic acid concentrations were determined by spectroscopy (Nanodrop ND-1000; Thermo Fischer Scientific).

### Antiviral and replicon assays

Transient replicon experiments and luminescence-based antiviral assays were performed in Huh7 cells as previously reported for the HEV Kernow-C1 p6/luc replicon (Debing et al., 2014b,c,d). α-interferon 2b (Intron-A) was purchased from Schering-Plough (Kenilworth, NJ). Ribavirin [1-(β-D-ribofuranosyl)-1H-1,2,4-triazole-3-carboxamide (Virazole)] was purchased from ICN Pharmaceuticals (Costa Mesa, CA). Mycophenolic acid was obtained from Santa Cruz Biotechnology (Dallas, TX) and sofosbuvir was from Selleckchem (Munich, Germany).

### *In vitro* rat HEV culture

Huh7 or HepG2/C3A cells were seeded in six-well plates at 2×10^5^ cells per well in 2 ml of DMEM with 10% FBS, penicillin (100 units/ml; Gibco) and streptomycin (100 g/ml; Gibco) and were incubated at 37°C for 24 h. For infection, 10% liver or feces suspensions were diluted 1:5 in DMEM with 10% FBS and filtered through a 0.2 µm filter. Culture medium was removed and cell layers were inoculated with 1 ml of the resulting solution (corresponding to approximately 2×10^8^ viral RNA copies). Plates were shaken for 1 h at room temperature and subsequently incubated at 35°C for 4 h. Afterwards, the inoculum was removed, cell layers were washed three times with 2 ml of PBS, and 2.5 ml of medium was added to each well. One ml of the medium was removed every 2-3 days, stored at −80°C and 1 ml of fresh medium was added to each well. After 20 days, cell layers were lysed and intracellular RNA was extracted with the Qiagen RNeasy kit. Viral RNA was extracted from culture medium with the NucleoSpin RNA virus kit (Macherey-Nagel, Düren) and quantified by reverse-transcription quantitative PCR (RT-qPCR).

To assess the *in vitro* replication of pLA-B350, Huh7 cells were seeded into six-well plates at 2×10^5^ cells per well and transfected 24 h later with capped RNA transcripts (1 µg per well) using Lipofectin (Life Technologies). Ribavirin was either not included in the culture medium or added to a concentration of 50 µM. One ml of medium was removed every 2-3 days, stored at −80°C and 1 ml of fresh medium (with or without ribavirin) was added to each well. After 20 days, cell layers were lysed and intracellular RNA was extracted with the Qiagen RNeasy kit. To extract viral RNA from culture medium, RNase A (Promega) was added to 150 µl of thawed medium to a final concentration of 200 ng/ml and incubated at room temperature for 5′ to reduce the amount of residual *in vitro* transcripts from RNA transfection. Viral RNA was extracted with the NucleoSpin RNA virus kit and quantified by RT-qPCR.

### Animal experiments

All animal studies were performed in accordance with local institutional guidelines and regulations, including approval for all animal experiments by the local Ethical Committee for Animal Experiments of KU Leuven, Belgium (project number: P007-2015). Five-week-old homozygous female athymic nude Hsd:RH-*Foxn1^rnu^* rats (*Rattus norvegicus*; Envigo, Horst, The Netherlands) were injected intravenously in the tail vein with 200 µl of a 1% liver homogenate of rat B350 (corresponding to approximately 2×10^7^ viral RNA copies, generated by homogenizing a liver fragment in PBS to obtain a 10% suspension that was subsequently diluted tenfold before injection) or injected intrahepatically with 5 µg of capped or non-capped pLA-B350 RNA (in 40 µl of H_2_O, spread over two or three injection sites). Feces were collected every 2-3 days, whereas serum was collected once a week through the tail-cut method; samples were stored at −80°C. For transmission experiments, rats were injected intravenously in the tail vein or intrahepatically with 200 µl of rat B350 liver homogenate and infected animals were co-housed with non-infected sentinels (*n=*3 for each group) for 3 weeks. Cage bedding was changed every week. Every morning, sentinels were fasted for 2 hours. For antiviral experiments, ribavirin was dissolved in PBS at 30 mg ml^−1^ and animals were injected intraperitoneally with 1 µl PBS or ribavirin solution per g body weight once daily (final dose: 30 mg kg body weight^−^^1^ day^−1^) for 14 days. At the indicated time points, animals were sacrificed by intraperitoneal pentobarbital injection, and serum and liver tissue were collected. For fecal samples, 10% suspensions in PBS were prepared by vortexing. After centrifugation, supernatant was used for either infection of cell cultures or for RNA extraction using the NucleoSpin RNA virus kit. RNA was extracted directly from serum samples in the same way. For liver samples, 10% suspensions were prepared through homogenization in PBS for infection of cell cultures or in buffer RLT for subsequent RNA extraction with the Qiagen RNeasy kit.

### RT-qPCR

For TaqMan-based quantification of rat HEV RNA, the forward primer 5′-ATGGTGCTTTTATGGCGATTG-3′ and the reverse primer 5′-CAAACTCACTGAAATCATTCTCAAAAAC-3′ were used (Purcell et al., 2011). The probe was labeled with 6-carboxyfluorescein (FAM) at the 5′ end, ZEN internal quencher in the middle and Iowa Black Fluorescent Quencher (IBFQ) at the 3′ end (5′-FAM-TATGTTCAG-ZEN-GAGAAGTTGGAAGCCGCTGT-IBFQ-3′; Integrated DNA Technologies, Coralville, Iowa). Reactions were performed with One-Step qRT-PCR mix (Eurogentec, Seraing, Belgium) in a final volume of 25 µl containing 900 nM of each primer, 250 nM of probe and 5 µl of RNA sample, using an ABI 7500 Fast real-time PCR system (Applied Biosystems, Foster City, CA) under the following conditions: 30 min at 48°C and 10 min at 95°C, followed by 40 cycles of 15 s at 95°C and 1 min at 60°C. Data were analyzed with ABI Prism 7500 SDS software (version 1.3.1; Applied Biosystems). For absolute quantification, standard curves were generated using tenfold dilutions of the cloned target cDNA.

### Serology and histopathology

Serum ALT, AST and γGT levels were determined with the Roche/Hitachi Cobas c 701/702 analyzer. For liver histological examination, tissue samples were harvested at the indicated time points and subsequently fixed in 8% formaldehyde, embedded in paraffin, sectioned and stained with hematoxylin-eosin as previously described (Leyssen et al., 2003). The following parameters were evaluated for inflammation and liver injury: infiltration of portal tracks by lymphocytes and polymorphonuclear leukocytes, ballooned hepatocytes, acidophilic body formation and the interlobular necrosis of hepatocytes.
